# On the Defect Structure of Biaxial Nematic Droplets

**DOI:** 10.1038/s41598-018-20492-0

**Published:** 2018-02-01

**Authors:** C. Chiccoli, L. R. Evangelista, P. Pasini, G. Skačej, R. Teixeira de Souza, C. Zannoni

**Affiliations:** 1 INFN Sezione di Bologna, Via Irnerio 46, 40126 Bologna, Italy; 2 Departamento de Física, Universidade Estadual de Maringá, Avenida Colombo, 5790-87020-900 Maringá Paraná, Brazil; 3 Faculty of Mathematics and Physics, University of Ljubljana, Jadranska 19, SI-1000 Ljubljana, Slovenia; 4 Departamento Acadêmico de Física, Universidade Tecnológica Federal do Paraná, Campus Apucarana, Rua Marcílio Dias, 635 CEP 86812-460 Apucarana Paraná, Brazil; 5 Dipartimento di Chimica Industriale “Toso Montanari”, Università di Bologna and INSTM, Viale Risorgimento 4, I-40136 Bologna, Italy

## Abstract

We present a detailed Monte Carlo study of the effects of molecular biaxiality on the defect created at the centre of a nematic droplet with radial anchoring at the surface. We have studied a lattice model based on a dispersive potential for biaxial mesogens [Luckhurst *et al*., Mol. Phys. 30, 1345 (1975)] to investigate how increasing the biaxiality influences the molecular organisation inside the confined system. The results are compared with those obtained from a continuum theory approach. We find from both approaches that the defect core size increases by increasing the molecular biaxiality, hinting at a non universal behaviour previously not reported.

## Introduction

It is well known that a sufficiently large nematic droplet embedded in a host matrix with perpendicular (homeotropic) alignment at the interface presents a defect at the centre of the system^1^. Dispersions of minute nematic droplets in a polymer matrix, e.g. of micron - size in Polymer Dispersed Liquid Crystals, PDLC^2^, or of nano-size droplets in H-PDLC^3^, the variant for holographic masks, are technologically important for their applications in optics. The shape and size of this defect core region has been extensively studied by a number of authors over many years^1–19^. For instance, the true point-like or ring-like nature of the core has been debated^20^. Recently, this problem has been the object of a renewed interest^17,18^, also using molecular level, off-lattice, simulations^21^, like in the work of de Pablo and his group^22,23^, but the majority of the studies concerns uniaxial nematics formed of uniaxial particles, notwithstanding the fact that, even if biaxial nematics^24^ are still rare, the majority of mesogens is not uniaxial and would be better represented by biaxial objects. Some theoretical speculations regarding nematic droplets formed by biaxial molecules have been put forward^4,15^, but computer simulations tackling this problem have been scarce (see, however^25^). Here, we aim to study, by means of a Monte Carlo approach, how the deviation from the cylindrical symmetry of the constituent molecules affects the defect core at the center of the droplet.

## The Simulation Model

We deal with a discretised version of the orientational biaxial potential of dispersive nature put forward many years ago by Luckhurst *et al*.^26^, and whose phase diagram for bulk systems has already been studied in detail by some of us, with Monte Carlo computer simulations^27,28^. This lattice model reproduces the rich phase diagram of a biaxial nematic system with isotropic, uniaxial, and biaxial phases, and it reduces to the well known Lebwohl-Lasher^29^ uniaxial model for nematics when the molecular biaxiality vanishes. The biaxial model Hamiltonian that we employ here consists of two parts, the first representing the interaction between the mesogenic molecules and the second the surface interactions between mesogens and the particles representing the surrounding media, endowed with a fixed orientation suitable to impose the desired boundary conditions:

1 $${U}_{N}=\frac{1}{2}\sum _{i,j\,\in \, {\mathcal F} i\ne j}{{\rm{\Phi }}}_{ij}+J\sum _{i\,\in \, {\mathcal F} j\,\in \,{\mathscr{J}}}{{\rm{\Phi }}}_{ij},$$

where ${\mathcal F}$, ${\mathscr{J}}$ are the set of particles (“biaxial spins”) in the bulk and at the surfaces, respectively, and the parameter *J* expresses the strength of the coupling with the surface particles, which is assumed to be a constant for a given substrate. The assumption is a reasonable one, as atomistic simulations of the nematic 5CB on different surfaces, like silicon^30^ and silica^31^, have shown that the order of the nematic at the surface and the anchoring orientation are strongly dependent on the chemical composition, morphology and roughness of the substrate but not on temperature, being very similar even in the isotropic and nematic phase.

The model is a purely orientational one and the spins are assumed to be at the sites of a cubic lattice and to interact by means of the second rank attractive pair potential derived from dispersive interactions as described in detail in:^26^

2 $${{\rm{\Phi }}}_{ij}=-{\varepsilon }_{ij}\{{P}_{2}(\cos \,{\beta }_{ij})+2\lambda [{R}_{02}^{2}({\omega }_{ij})+{R}_{20}^{2}({\omega }_{ij})]+4{\lambda }^{2}{R}_{22}^{2}({\omega }_{ij})\},$$

where *ε*_*ij*_ is a positive constant, *ε*, for nearest neighbour molecules *i* and *j*, and zero otherwise; *ω*_*ij*_ is the relative orientation of the pair of spins, given by three Euler angles (*α*, *β*, *γ*)^32^ and ${R}_{mn}^{L}$ are combinations of Wigner functions symmetrised for a *D*_2*h*_ biaxial phase^27^. The biaxiality parameter *λ* takes into account the deviation from cylindrical molecular symmetry and *λ* ≠ 0 indicates that the particles tend to align not only their major (“long”) axis, but also their short (“transversal”) axes. Notice that both the mesogens and the boundary particles are taken to be biaxial, with the same dispersive functional form Φ_*ij*_.

## Simulation and Results

We have investigated biaxial droplets with radial boundary conditions for various values of molecular biaxiality. All the simulations for the model droplet have been performed on approximately spherical samples (our droplets) carved from a 50 × 50 × 50 cubic lattice and containing 54474 particles. The parameter *J*, denoting the surface coupling with the surrounding environment, is taken equal to one, which means that the interaction between the molecules of the nematic and those of the host material surrounding the embedded droplet has the same strength of the mesogen-mesogen interaction. To simulate the optical texture we have employed the Stokes-Muller methodology described in^33–35^ and the following parameters, reported to real units: droplet diameter *d* = 5.3 *μ*m, and, assuming that the refraction tensor can always be considered as approximately uniaxial, with ordinary and, extraordinary refractive indices *n*_*o*_ = 1.5 and *n*_*e*_ = 1.66, for a light wavelength *λ*_*o*_ = 545 nm.

We have investigated the cases in which the molecules at the droplet surface have the long axis radially oriented, while the short axes have random orientation in the locally tangent plane. The temperature was set to the dimensionless value T^*^ = *k*_*B*_*T*/*ε* = 0.1, deep in the ordered phase.

**Figure 1:**
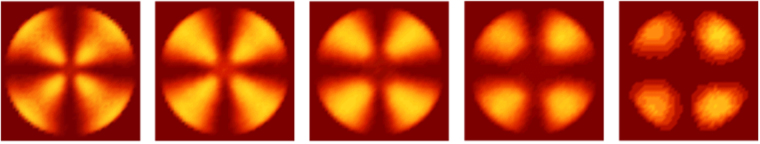
Optical texture obtained from MC simulation of a droplet with RBC between crossed polarizers for various values of the molecular biaxiality *λ* = 0.10, 0.15, 0.20, 0.25, 0.30 (from left to right).

**Figure 2:**
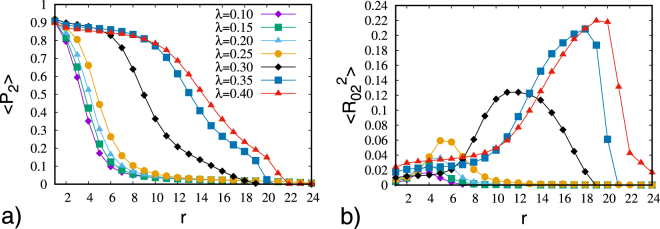
Second rank order parameters < *P*_2_ > (**a**) and $< {R}_{02}^{2} >$ (**b**) versus distance starting from the center of the droplets.

**Figure 3:**
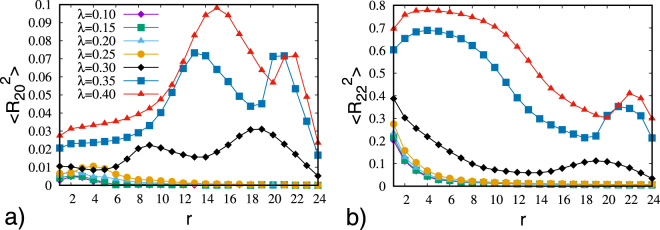
Second rank biaxial order parameters $< {R}_{20}^{2} >$ (**a**) and $< {R}_{22}^{2} >$ (**b**) versus distance starting from the center of the droplets.

The results, reported in Fig. 1, show that the pattern changes from a configuration similar to the uniaxial case, where at the centre of the droplet a four leaves pattern consistent with a point defect is present, to a final pattern, where the defect core tends to increase. These features can be quantitatively confirmed by looking at the ordering inside the droplet. The four second rank order parameters typical of a biaxial phase were calculated starting from the centre of the droplet and going towards the surface. The results are presented in Figs 2 and 3. We see that the central region corresponds to a small well ordered domain, as seen in the uniaxial case^11^.

**Figure 4:**
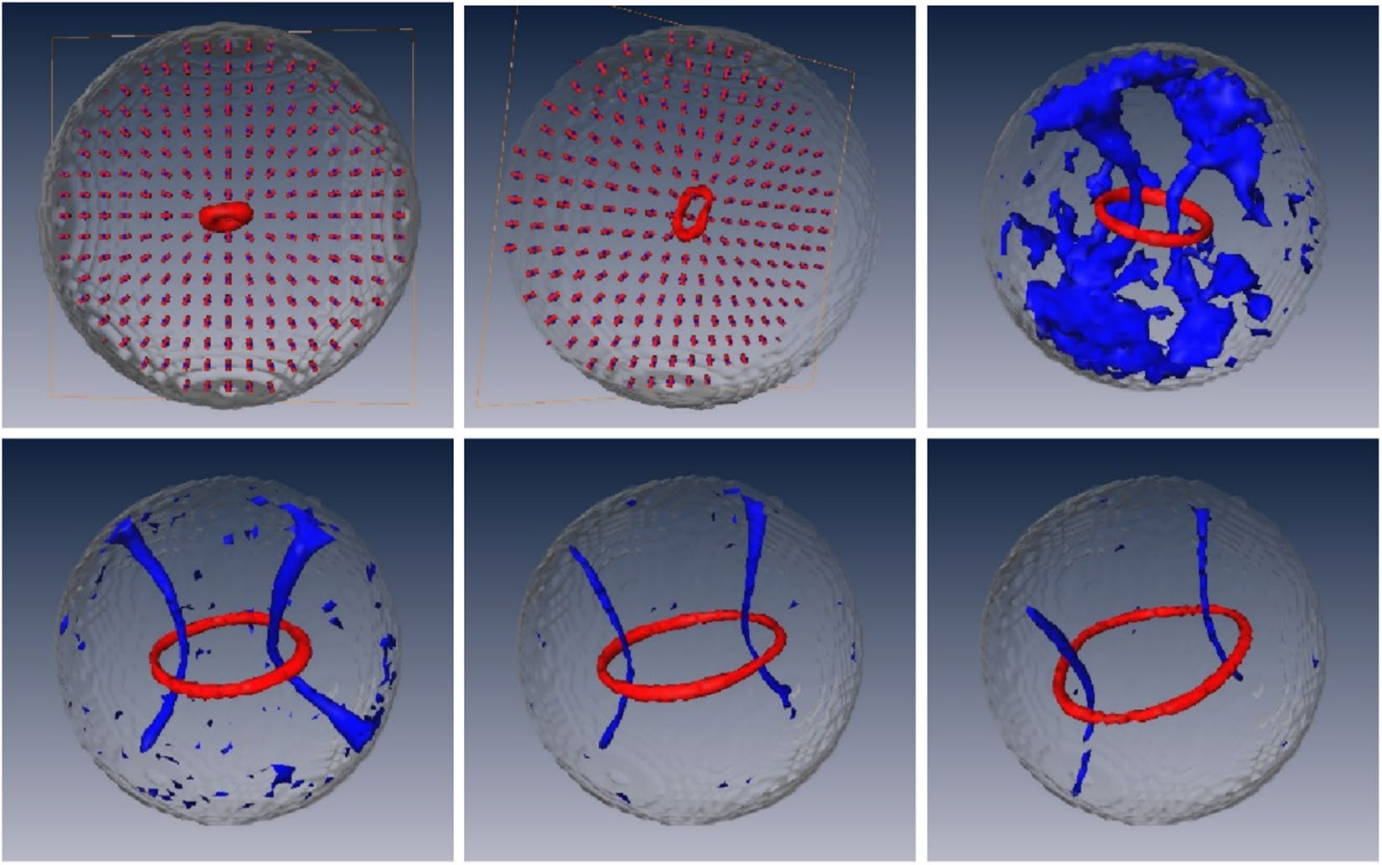
Plots of the *c*_*l*_ Westin metric isosurfaces^36^ for the principal and secondary director (red and blue, respectively). The isosurface thresholds vary from plot to plot in order to provide an optimal visualization of areas where the directors are not well defined. The images are for *λ* = 0.10, 0.20, 0.30, 0.325, 0.35, 040 (from top left to bottom right).

It is clear from Fig. 2 that the extension of the ordered core increases as the molecular biaxiality increases. Moreover, the core of the defect is biaxial^13^, as confirmed by looking at the biaxial order parameters (see Fig. 3). The enlargement of the radius of the defect core as the molecular biaxiality increases can be visualized by plotting the isosurfaces for the values of < *P*_2_ >, as shown in Fig. 4. It can be noticed that when *λ* approaches the value of 0.3 we have a conspicuous change in the scenario. In fact, two disclinations start to appear related to the second molecular axis, which are clearly evident for larger molecular biaxiality.

## Continuum Theory Approach

A full analysis from the elastic continuum theory point of view (see, e.g^37^) is not easily approached, if we consider that calculating the bulk free energy for biaxial nematic liquid crystals requires twelve elastic constants and three surface terms^38^. These constants are associated to the deformations of splay, twist, and bend of the triad of vectors of the axes of the molecules, and other coupling constants connected to these three axes^39,40^. However, following the arguments presented in the work of Sukumaran and Ranganath^15^, it is possible to write the free energy in a simplified way, somehow in the same spirit of the classical one-constant approximation of uniaxial nematics, in which the coupling constants can be neglected and the constants of splay, twist, and bend, associated to the same axes, can be considered alike. This procedure reduces from twelve to three the number of elastic constants, and the free energy can be written as:

3 $$\begin{array}{rcl}f & = & \frac{{K}_{a}}{2}[{(\overrightarrow{a}\cdot \nabla \overrightarrow{b}\cdot \overrightarrow{c})}^{2}+{(\overrightarrow{b}\cdot \nabla \overrightarrow{b}\cdot \overrightarrow{c})}^{2}+{(\overrightarrow{c}\cdot \nabla \overrightarrow{c}\cdot \overrightarrow{b})}^{2}]\\  &  & +\frac{{K}_{b}}{2}[{(\overrightarrow{b}\cdot \nabla \overrightarrow{c}\cdot \overrightarrow{a})}^{2}+{(\overrightarrow{c}\cdot \nabla \overrightarrow{c}\cdot \overrightarrow{a})}^{2}+{(\overrightarrow{a}\cdot \nabla \overrightarrow{a}\cdot \overrightarrow{c})}^{2}]\\  &  & +\frac{{K}_{c}}{2}[{(\overrightarrow{c}\cdot \nabla \overrightarrow{a}\cdot \overrightarrow{b})}^{2}+{(\overrightarrow{a}\cdot \nabla \overrightarrow{a}\cdot \overrightarrow{b})}^{2}+{(\overrightarrow{b}\cdot \nabla \overrightarrow{b}\cdot \overrightarrow{a})}^{2}]\mathrm{.}\end{array}$$

In Eq. 3, $\overrightarrow{a}$, $\overrightarrow{b}$ and $\overrightarrow{c}$ are the orientations of the three unit vectors of the biaxial director frame, and the constants *K*_*i*_ are the elastic constants associated to each direction. This free energy reduces to the one of the uniaxial case if the replacement $\overrightarrow{c}\to \overrightarrow{n}$ can be performed in addition to assuming *K*_*c*_ = 0 and *K*_*a*_ = *K*_*b*_. In the limit of weak biaxiality, it is possible to consider *K*_*a*_ ≈ *K*_*b*_ and to reduce to two the number of elastic constants. For future purposes, let us define *k*_*ac*_ = *K*_*c*_/*K*_*a*_. This quantity seems to be presumably related to the biaxiality parameter *λ* considered in the pair potential Eq. 2. Indeed, *λ* is connected with the deviation from uniaxiality of the molecule^18^. On the other hand, from a pseudomolecular point of view, the elastic constants of a uniaxial nematic are also dependent on the anisometric shape of the molecular building blocks. In the limiting case of *λ* = 0, i.e. for the Lebwohl-Lasher model, only one elastic constant is obtained. Thus we can assume that the quantities *k*_*ac*_ and *λ* play an analogous role, as we will discuss later.

Due to the spherical symmetry, inside the droplet the triad of vectors can be written as

4 $$\begin{array}{rcl}\overrightarrow{a} & = & \sin \,[\xi (r,\theta ,\varphi )]\,\cos \,[\zeta (r,\theta ,\varphi )]\hat{r}\\  &  & +\,\cos \,[\xi (r,\theta ,\varphi )]\,\cos \,[\zeta (r,\theta ,\varphi )]\hat{\theta }\\  &  & +\,\sin \,[\zeta (r,\theta ,\varphi )]\hat{\varphi };\\ \overrightarrow{b} & = & -\,\sin \,[\xi (r,\theta ,\varphi )]\,\sin \,[\zeta (r,\theta ,\varphi )]\hat{r}\\  &  & -\,\cos \,[\xi (r,\theta ,\varphi )]\,\sin \,[\zeta (r,\theta ,\varphi )]\hat{\theta }+\,\cos \,[\zeta (r,\theta ,\varphi )]\hat{\varphi };\\ \overrightarrow{c} & = & \cos \,[\xi (r,\theta ,\varphi )]\hat{r}-\,\sin \,[\xi (r,\theta ,\varphi )]\hat{\theta },\end{array}$$

where *ξ* is the angle between $\overrightarrow{c}$ and the radial direction, while *ξ* is the angle of twist of $\overrightarrow{a}$ and $\overrightarrow{b}$ about $\overrightarrow{c}$.

The simulations suggest that a disclination loop is the stable configuration of the long axes of the biaxial director. By following the development of Kanke and Sasaki^17^ for uniaxial liquid crystals, the configuration for the long axis $\overrightarrow{c}$ can be approximately given by an oblate spheroid structure. In spherical coordinates, the angle *ξ* can be written as

5 $$\xi (r,\theta ,\varphi )=\theta -\arctan \{\frac{[{d}_{1}(r,\theta ,a)-{d}_{2}(r,\theta ,a)]}{[{d}_{1}(r,\theta ,a)+{d}_{2}(r,\theta ,a)]}\sqrt{\frac{{[{d}_{1}(r,\theta ,a)+{d}_{2}(r,\theta ,a)]}^{2}-1}{1-{[{d}_{1}(r,\theta ,a)-{d}_{2}(r,\theta ,a)]}^{2}}}\},$$

in which ${d}_{1,2}(r,\theta ,a)=\sqrt{{r}^{2}+{a}^{2}\pm 2ar\,\sin \,\theta }$ and *a* is the defect ring radius.

On the other hand, as discussed by Sukumaran and Ranganath^15^, the short axis could describe a disclination around the long axis, and, then,

6 $$\zeta (r,\theta ,\varphi )=-\varphi .$$

**Figure 5:**
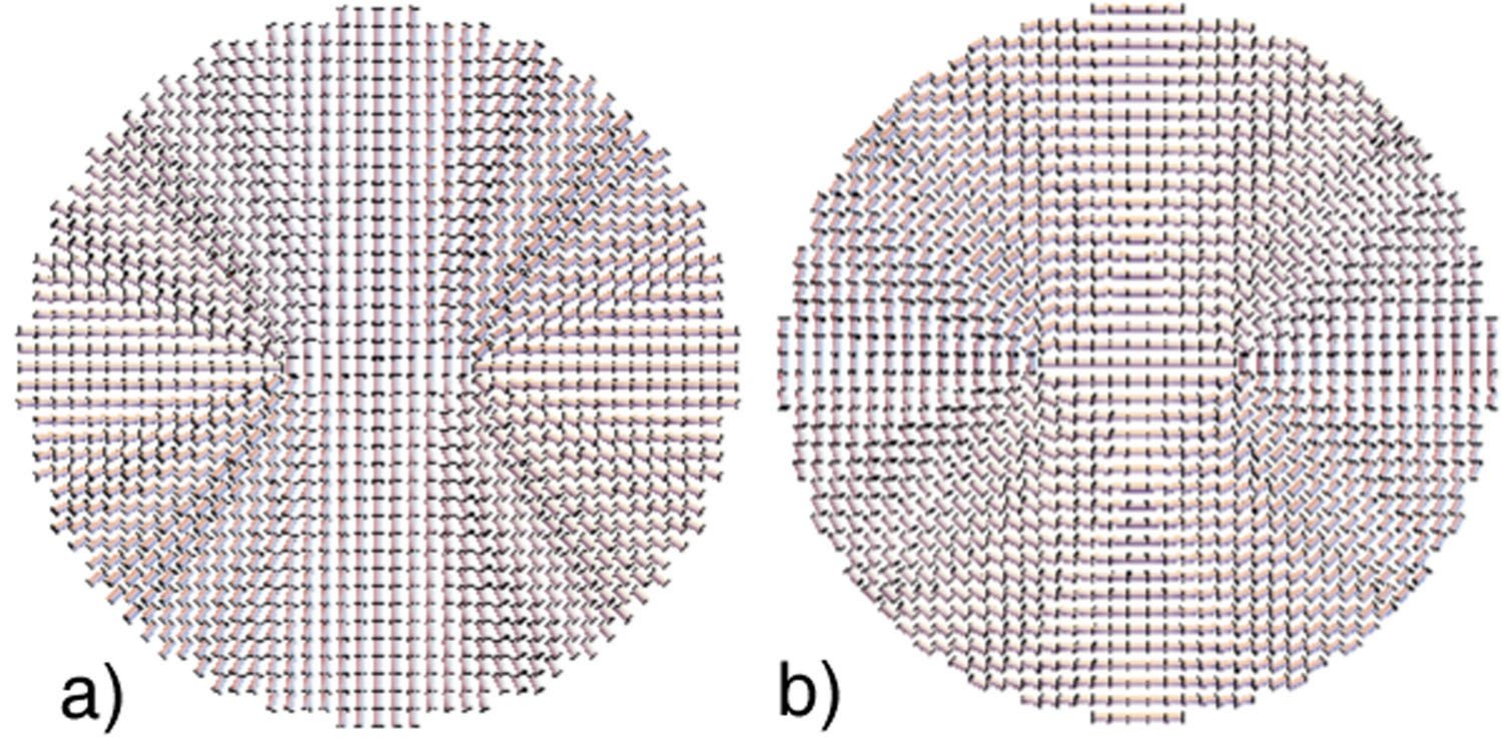
A cross section of the director configuration snapshot obtained from continuum theory: (**a**) long and (**b**) short axes in the *xz* plane of the droplet. The parameters used were the defect core radius *a* = 6.0 and the droplet radius *R* = 20. It is possible to observe that the proposed solution seems to reproduce quite well the defect structure observed in the simulations.

At the border of the droplet, these configurations do not guarantee the completely homeotropic configuration of the long axis and the planar alignment of the short axes; however, they yield a very good agreement with the results obtained by means of simulations, and they will be very useful to give us some qualitative insights about this system. A snapshot of these configurations can be individually observed in the Fig. 5a and b for the long and short axes, respectively.

**Figure 6:**
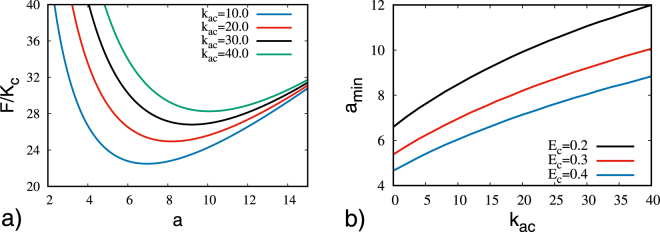
(**a**) Scaled energy as function of a hypothetical ring radius, presenting a minimum value for a specific value for *E*_*c*_ = 0.3. (**b**) The minimum value of energy, denoted as *a*_min_ as function of *k*_*ac*_ for some values of Ec showing that the defect ring core increases when the biaxial elastic constant increases.

By using these ansatzs, we numerically evaluate the free energy *F*_bulk_ by integrating (3) on the droplet volume and removing a small region around the ring defect. Thus, the energy is given by

7 $$F={F}_{{\rm{bulk}}}+2\pi a{E}_{c},$$

in which *E*_*c*_ is the defect core energy^17^. The results are presented in Fig. 6, in which we have used the radius of the droplet *R* of 20 units and removed a toroidal region of radius 0.2 units around the defect. This results confirm the analogy between *k*_*ac*_ and *λ* and indicate that they play a similar role in the changing of the dimension of the defect core. The simulation results show that the radius of the ring defect becomes larger as *λ* increases (see Fig. 4). Analogously, the minimum of the defect energy increases monotonically with *k*_*ac*_. Therefore, the anisometric shape of the molecule crucially influences the radius of the ring defects.

An analytical connection between the quantities *λ* and *k*_*ac*_ may be established by evaluating the energy of a given configuration in both the approaches, the elastic continuum theory and the discretized potential used in the computer simulation.

First, let us consider a specific triad of vectors, representing the biaxial director, in which the long axis is parallel to the *x*−direction, the second axis is parallel to *y*, and, consequently, the third axis is parallel to the *z*−direction. Then, at the point (0, 0, *L*), with *L* being a really small length, it is placed another molecule with the second and the third axes slightly tilted with respect to the first molecule by a small angle, *ζ* ≪ 1.

The discretisation process may be performed by considering the following triad of vectors:

$$\overrightarrow{c}=(1,0,0),\quad \overrightarrow{b}=(0,1,\zeta z/L),\quad {\rm{and}}\quad \overrightarrow{c}=(0,\zeta z/L,1).$$

Thus, the elastic free energy may be evaluated, and the result is approximated as

8 $$F={k}_{ac}{K}_{a}\frac{{\zeta }^{2}}{{L}^{2}}+{\mathscr{O}}({\zeta }^{4}).$$

Now, we consider an analogous situation with the biaxial particles. We assume that the Euler angles of two neighbouring molecules are given by (0, 0, 0) and (0, 0, *ξ*). The interaction between these two particles, by means of the biaxial pair potential, may be approximated as

9 $${\rm{\Phi }}=-4\varepsilon {\lambda }^{2}{\rm{Re}}[{e}^{2i\xi }].$$

If *ξ* ≪ 1, then (9) becomes

10 $${\rm{\Phi }}=-4\varepsilon {\lambda }^{2}+8\varepsilon {\lambda }^{2}{\xi }^{2}+{\mathscr{O}}({\xi }^{4}).$$

The result would be the same if the Euler angles of the particles were specified instead by (0, 0, 0) and (*ξ*, 0, 0). In this framework, the term ${k}_{ac}{K}_{a}\frac{{\zeta }^{2}}{{L}^{2}}$, in (8), can be compared with 8*ελ*^2^*ξ*^2^, in (10), in order to yield a direct relation connecting *k*_*ac*_ and *λ*^2^. Thus, the simple calculation presented above indicate that *k*_*ac*_ ∝ *λ*^2^ in a first approximation in which thermal fluctuations could be neglected.

Indeed, by analyzing the plot of the energy in Fig. 6a, it is possible to note a minimum value for a specific value of the ring radius, *a*, which we denote by *a*_min_. This value increases when the elastic constant *k*_*ac*_ increases, indicating that as the biaxiality becomes more evident, the defects radius becomes larger, as can be obtained from simulations. This result is confirmed when we analyze explicitly the profile of the *a*_min_ as a function of the biaxial elastic constant (Fig. 6b). Using this approach it is possible to note that, as already seen from the simulations, even in the limit of uniaxial liquid crystals (*k*_*ac*_ → 0) the system presents a ring defect structure, which is in agreement with the elastic theory for these materials^17^. It is interesting to notice that our results, even within an approximated approach, present a quite good qualitatively agreement with the simulations, in which the spins on the border are kept in a planar alignment of the director short axes, letting this increasing of the defect ring radius even more evident with the increasing of the biaxiality.

## Concluding Remarks

We have explored, by means of Monte Carlo simulations, how the deviation from cylindrical symmetry of the constituent molecules influences the defect core present at the center of a nematic liquid crystal droplet with radial boundary conditions for the longest axis. This has been done by using a lattice model based on a dispersive potential for biaxial mesogens, characterised by a molecular biaxiality parameter^26^. The core of the defect is biaxial and the simulations, keeping the long axis of the particles at the droplet surface radially oriented and the short one in a planar degenerate tangent alignment, confirm that the defect radius increases with the increasing of the molecular biaxiality. This result was reinforced by a continuum theory analysis based on the possibility of relating the biaxiality parameter of the pair potential with the ratio between some elastic constants. In such a system the number of elastic constants is reduced when the molecular biaxiality is low. It is shown that the minimum of the defect energy increases monotonically with this ratio, indicating again that the anisometric shape of the molecules, measured by this quantity, crucially affects the radius of the ring defects. In the limit of uniaxial liquid crystals, for which this ratio vanishes, the system continues to present a small and well defined ring defect structure, as expected from the elastic theory and computer simulations on these materials. The finding that the core defect radius depends not only on the rank of the interaction, as it has been observed for the Heisenberg and Lebwohl-Lasher models for magnetic and nematics interactions^11^ but also on an apparent molecular detail, such as the mesogen biaxiality, is showing a non-universal behaviour that, as far as we know, has not been reported before and that should stimulate further theoretical and experimental investigations.
